# Messaging Circumstances and Economic Pressures as Influences on Linkage to Medical Male Circumcision following Community-Based HIV Testing for Men in Rural Southwest Uganda: A Qualitative Study

**DOI:** 10.1155/2018/8387436

**Published:** 2018-05-03

**Authors:** Hannah N. Gilbert, Monique A. Wyatt, Stephen Asiimwe, Bosco Turyamureeba, Elioda Tumwesigye, Heidi Van Rooyen, Ruanne V. Barnabas, Connie L. Celum, Norma C. Ware

**Affiliations:** ^1^Department of Global Health and Social Medicine, Harvard Medical School, Boston, MA, USA; ^2^Harvard Global, Cambridge, MA, USA; ^3^Integrated Community Based Initiatives, Kabwohe, Uganda; ^4^Human Sciences Research Council, Durban, South Africa; ^5^School of Clinical Medicine, Faculty of Health Sciences, University of the Witwatersrand, Johannesburg, South Africa; ^6^Department of Global Health, School of Medicine and School of Public Health, University of Washington, Seattle, WA, USA; ^7^Division of Global Health Equity, Department of Medicine, Brigham and Women's Hospital, Boston, MA, USA

## Abstract

Voluntary medical male circumcision (MMC) reduces risk of HIV infection, but uptake remains suboptimal among certain age groups and locations in sub-Saharan Africa. We analysed qualitative data as part of the Linkages Study, a randomized controlled trial to evaluate community-based HIV testing and follow-up as interventions promoting linkage to HIV treatment and prevention in Uganda and South Africa. Fifty-two HIV-negative uncircumcised men participated in the qualitative study. They participated in semistructured individual interviews exploring (a) home HTC experience; (b) responses to test results; (c) efforts to access circumcision services; (d) outcomes of efforts; (e) experiences of follow-up support; and (f) local HIV education and support. Interviews were audio-recorded, translated, transcribed, and summarized into “linkage summaries.” Summaries were analysed inductively to identify the following three thematic experiences shaping men's circumcision choices: (1) intense relief upon receipt of an unanticipated seronegative diagnosis, (2) the role of peer support in overcoming fear, and (3) anticipation of missed economic productivity. Increased attention to the timing of demand creation activities, to who delivers information about the HIV prevention benefits of MMC, and to the importance of missed income during recovery as a barrier to uptake promises to strengthen and sharpen future MMC demand creation strategies.

## 1. Introduction

Linkage to antiretroviral treatment and effective prevention following widespread testing promises to reduce HIV incidence in Africa. A number of strategies to increase linkage are being evaluated, including integration and “streamlining” of services, point of care CD4 and viral load testing, economic incentives, and community-based testing with counselling and follow-up support [1–6]. Active linking to prevention services, such as voluntary medical male circumcision (MMC) is an important component of this comprehensive approach.

The efficacy of voluntary medical male circumcision (MMC) in reducing the risk of HIV infection has been demonstrated in three randomized controlled clinical trials (RCTs) in Africa. These RCTs revealed a nearly 60% reduction in female-to-male HIV transmission [7–9]. On the basis of this evidence, the World Health Organization and the Joint United Nations Program on HIV/AIDS (WHO/UNAIDS) recommended MMC as an effective component of combination prevention strategies in key countries with generalized heterosexual epidemics exhibiting both high rates of HIV and low rates of male circumcision [10].

Despite evidence of effectiveness, uptake of MMC has been suboptimal among African men in certain locations and age groups. According to 2015 figures, Uganda achieved 64% progress towards the 80% coverage target set by the WHO in 2011. The WHO has established a new 2020 Fast Track target of 90% MMC coverage. As funding gaps for MMC have increased across sub-Saharan Africa, the WHO has called for new and innovative approaches to make MMC programs more efficient [11]. MMC research priorities are increasingly oriented towards implementation, with a specific focus on how to generate interest and demand for the procedure [12–14]. Studies of MMC implementation programs have identified barriers associated with circumcision uptake; new analyses target incentives for men to pursue circumcision despite the obstacles they face [15].

The Linkages Study was a randomized controlled trial assessing the effectiveness of community-based HIV testing and counselling with follow-up support in linking HIV-positive individuals to HIV care and HIV-negative uncircumcised men to MMC. The trial was carried out in rural KwaZulu-Natal, South Africa, and in Kabwohe, Sheema District, in rural southwest Uganda. Based upon qualitative data collected as part of the Linkages Study, this paper describes key experiences impacting uncircumcised Ugandan men's interest in and ability to pursue MMC following a negative HIV test result. From these experiences, we draw inferences aimed at informing creation of demand for MMC services and strengthening strategies to promote linkage to HIV prevention in rural African settings.

## 2. Methods

### 2.1. The Linkages Study

Linkages Study participants received community-based HIV testing and counselling at home or via a mobile van. Individuals testing positive and not already prescribed antiretroviral therapy (ART) were randomized to one of two follow-up support interventions, or a control condition. Uncircumcised men testing negative were randomized to follow-up support interventions intended to stimulate demand for MMC: (1) home visits from lay counsellors at 1 and 3 months, (2) or text message reminders. The control condition was promotion of MMC at the testing visit. Men received text messages three weeks after testing, and follow-up calls at one month. Those reporting not having been circumcised received a second text message 6-7 weeks after testing, and a second follow-up call.

Uptake of circumcision in the Linkages Study was 42% (52/123) (RR = 2.37, 95% CI: 1.55, 3.63) for men receiving lay counsellor follow-up support, compared to 18% (23/129) for controls and 33% (52/156) for men receiving follow-up text messages compared to controls (RR = 1.87, 95% CI: 1.21, 2.88). Additional information on intervention results may be found elsewhere [5].

### 2.2. Sampling and Recruitment for the Qualitative Research

A purposeful sampling approach aims to maximize information-rich cases and capture variation in order to provide in-depth information about the topic under study [16]. To maximize variation, the qualitative study adopted a purposeful sampling approach that aimed to identify participants at different points across the trial follow-up period, targeting experiences of home HTC and follow-up support in linkage to HIV treatment, care, and MMC prevention services.

Potential participants were sampled at 1–3 months, 8–12 months, or >12 months following enrolment in the Linkages Study to ensure representation of perspectives from participants in both the first months and the later months of follow-up (see Table 1). The qualitative sample included two subgroups: 47 individuals testing HIV-positive and assigned to follow-up support or control for linkage to HIV treatment and care and 52 uncircumcised men testing negative and assigned to follow-up or control for linkage to MMC. This analysis draws exclusively on data from the subgroup of 52 uncircumcised Ugandan men.

Linkages experiences of the HIV-positive, treatment group in Uganda have been described by Ware and authors [17]. Qualitative and quantitative Linkages Study results from South Africa have been published elsewhere [18–20].

### 2.3. Data Collection

Data collection for the qualitative study included individual, semistructured interviews. These interviews relied on an interview guide consisting of open-ended questions that explored the following topics: (a) home HTC experience; (b) responses to test results; (c) plans to access HIV care or MMC prevention services; (d) outcomes of plans; (e) experiences of follow-up support; and (f) HIV education and support in local communities. Ugandan research assistants (RAs) trained in qualitative methods conducted the interviews in private settings, in the local language. Interviews took place from December 2013 through March 2015. They lasted 45–60 minutes and were audio-recorded. This analysis draws on interviews with 52 men who were seronegative and uncircumcised at the time of enrolment; it includes the experiences of men who ultimately went on to be circumcised and those who did not.

### 2.4. Data Quality

Audio-recordings were transcribed in English by the RAs. Transcripts were reviewed for quality as they were completed and used to provide continuous feedback on interview content and technique.

### 2.5. Data Analysis

Data analysis relied on an inductive, content analytic approach to develop thematic concepts [21]. Analysis began with data reduction. To reduce the data, two members of the research team read through the interview transcripts to create a “linkage summary” for each interviewee. These summaries recapitulated the interviewee's HIV testing and linkage experiences as they described them to highlight the individual trajectories involved and served in lieu of coding as a data reduction technique [16]. Summaries were subjected to thematic analysis. The documents were reviewed for repeated content to identify emergent patterns in interviewees' testing and linkage experiences. Employing an iterative process, these patterns were formulated as summary statements. The statements summarize themes resulting from the analysis, to represent key aspects of interviewees' experiences of HIV testing and perspectives on male circumcision, in terms of their impact upon subsequent linkage to services. As such, they constitute the key findings of the analysis. Summary statements appear as 1–3 in Results. Each is elaborated using descriptive content from the interviews and illustrated through quotes from interviewees.

### 2.6. Ethical Statement

The qualitative study was reviewed by the Committee on Human Studies at Harvard Medical School, the Human Subjects Division of the University of Washington, and the Uganda National Council for Science and Technology. Participants provided consent for the qualitative interviews as part of the Linkages Study consent process. Consent was reconfirmed verbally for the qualitative study.

## 3. Results

### 3.1. Characteristics of Participants

Median age of men taking part in the qualitative study was 26.5 (IQR 22.75–32.25) at Linkages Study enrolment. Slightly more than half (54%) were married. Forty percent (*N* = 21) had been randomized to counsellor follow-up for linkage to MMC services following home HIV testing; 54% were assigned to follow-up through text messages. Half were interviewed 12 months or more into follow-up. At exit, 42%  (*N* = 22) of men taking part in the qualitative study had been circumcised.

### 3.2. Qualitative Results

The qualitative analysis revealed three themes in these men's experiences of linking (or not linking) to circumcision services, as described below.

#### 3.2.1. Relief at Unexpectedly Receiving a Negative HIV Test Result Created Strong Motivation to Link

Many men reported entering the home HIV testing encounter convinced they would receive a seropositive diagnosis. Some believed they were HIV-positive because HIV was perceived as a ubiquitous, ever-present threat. Others felt at risk because they had engaged in unprotected sex with multiple partners, or with a partner presumed to be HIV-positive. Many offered persistent STI infections as presumed “proof” of seropositivity, with one many describing themselves as “a daily customer of gonorrhoea.”

When provided with a negative test result, these men were shocked and elated. The experience was described as a “second chance” at life—*“like I had gone to heaven… received a new life*,*”* in the words of one. The intense relief associated with this “second chance” translated into a determination to preserve health for the future. One man described his experience this way.

I felt very happy and loved my life again. I loved my life and made good plans… you know, when you don't expect to be negative and you find you are negative you have all the reasons to love your life and make good plans. (Male, age 40)

This surprise and delight instilled in participants a resolve to stay HIV-negative. Following the Linkages Study protocol, counsellors who delivered test results immediately followed up with messages about HIV prevention benefits of circumcision. With newfound determination to avert future infection, men were eager to take steps towards preserving their health. Circumcision presented an immediate and feasible means of enacting “good plans” to remain uninfected. The following quotes illustrate:

When the counsellor tested me and I found that I don't have HIV I became happy. When they convinced me that I should circumcise because the cover can keep viruses after having sex, [then] I decided to circumcise as one way of protecting myself against HIV. (Male, 38 years old)

When he [counsellor] finished testing, he told me that I don't have HIV virus in my blood and I praised God. He told me that I have to make sure to protect myself against the HIV virus. Now that I know I am HIV–negative, I shouldn't do wrong things. At that time I told him that I want to circumcise. (Male, age 49)

#### 3.2.2. Hearing Circumcision Success Stories from Peers Helps Overcome Fear of the Procedure

Fear emerged as a barrier to circumcision in this study. Some men feared physical pain—what one participant called* “the pain inflicted by the circumcision knife*.*”* Expected invasiveness of the surgery was particularly concerning for others.

Me, I thought they would cut me deeply… Like cutting the whole penis. I thought that they were going to cut like a part and throw it away. (Male, age 24)

The emotional and marital impact of refraining from sexual intercourse during convalescence was a circumcision-related fear for many. Still others worried that the procedure might permanently compromise their sexual health.

I am worried that if I circumcise I will be sexually weak… because they will cut some parts of my manhood which God created with me [sic]. And I think that will make me sexually weak in the future. (Male, age 19)

Some men were able to overcome their fears and follow through on their resolve to circumcise. Support from peers who had successfully completed the procedure emerged as the lynchpin enabling men to act upon their initial receptivity to circumcision. Peer support included reminders, transport, and accompaniment to the clinic. The most widely acknowledged form of support, however, was positive reports from circumcised peers, whose experiences counteracted circumcision fears. One young man described his brother's influence.

Since he was already circumcised, he told me that circumcision prevents you from many diseases, especially STDs. He also told me that the pain is not much and even said he would volunteer to take me to the Health Centre. (Male, age 24)

Another man explained that he often bathes in the river with his friends, where the topic of circumcision is frequently raised.

We do the same work so sometimes, we may be discussing and such topics come [up], and they tell me [about circumcision]. We even bathe from the same river here in the village, so I see them… They like it [circumcision] and they encourage me to do it...like telling me to go and circumcise and telling me their experiences. (Male, age 49)

The effectiveness of these success stories as a circumcision driver was dependent upon identifying relevant “peers.” Peers were local friends, neighbours, or family members, who, importantly, were similar in age.

Our people here… you see if one is not of your age, he can't listen to you. The person you can tell is someone of your age. Someone who is in the same age bracket as you. But for people who are older, at times, they don't even need your advice. For them, they don't want that. (Male, age 30)

#### 3.2.3. Anticipation of Missed Economic Productivity Was an Insurmountable Barrier to Linkage for Many Men

Many men who emerged from the testing encounter keen to pursue circumcision explained that they were unable to follow through because they anticipated a healing period that could last weeks. The economic consequences of this period of missed work were particularly significant for men who performed agricultural or day labour for income. They explained that their work consisted of planting, cutting, and carrying grasses or transporting bananas on bicycles; they relied entirely upon their physical strength for survival. The unrelenting schedule of agricultural labour afforded no “down time” to recover, as the following examples illustrate:

I dig; okay, the most important thing is digging. I use my hands, so because I have to work to eat, you find that I have no time to get circumcised. (Male, age 22)

I do manual work with my hands, which means if I don't work I will not be having what to eat. That's what I fear… but I like circumcision. (Male, age 39)

The significance of missed productivity was compounded by men's positions as family providers. Men with multiple dependents faced a formidable set of daily responsibilities that forced them to weigh the long-term health benefit of increased HIV protection against the immediate food and economic needs of their families. In such a calculus, circumcision was simply not an option.

I realized that if I go and circumcise and sit home without working, my family will starve. I will not be having money since I will not be working. If I spend time at home not working during the recovery period, my family cannot survive, because they will need home items yet I will not be having money. (Male, age 27)

The few men who overcame this economic barrier had more flexibility in their social and economic lives. These men held jobs outside of the agricultural sector and could shift their workload to accommodate recovery time. For example, a policeman scheduled his circumcision between demanding “special assignments” when he could do light deskwork.

As I was not doing a lot of work, I would wake up, bathe, put on my uniform, go and sit at the bank where I was working as a security personnel and work. (Male, age 38)

These men were also often unmarried and thus not financially responsible for a family. Their expenses were straightforward and predictable, allowing them to calculate precisely how much money would be required to support themselves during their convalescence

Okay, I had prepared in advance because I knew that I was going to get circumcised. They had said you can get a problem and spend days without working. Because of that, I kept some money aside to use while waiting to recover. (Male, age 25)

## 4. Discussion

Many men in this study entered the home testing encounter expecting a positive diagnosis; the unanticipated news that they were HIV-negative created a strong motivation to preserve health. Some men then simply went on to be circumcised. For others, initial motivation was stymied by fears about the procedure. Circumcision success stories, a set of informal communications in which circumcised men described positive experiences of the procedure, helped some men overcome their fear. Others, while motivated to circumcise, were unable to act because of the economic challenges posed by missed work. These men were unable to reconcile the long-term health benefits of circumcision with the short-term imperatives of providing for their families in an economy based on agricultural labour. These men saw missed productivity during the MMC recovery period as an insurmountable barrier.

A number of lessons may be drawn from these findings to inform demand creation for MMC. One such lesson has to do with the timing of demand creation activities. For men participating in this study, relief and joy at learning their HIV test result was negative quickly evolved into new determination to remain free of HIV. We propose this new determination represented a moment of particular receptivity to receipt of education and counselling about the HIV prevention benefits of circumcision, suggesting both that the timing of demand creation interventions makes a difference and that optimal timing coincides with “moments” when men may be predisposed to hearing and acting on HIV prevention information. One such moment, this study teaches us, is immediately after receiving a negative HIV test result.

A second lesson for MMC demand creation that may be drawn from these study results speaks to the question of who should deliver information about the prevention benefits of circumcision. Our study and others [22, 23] suggest that peers who have been through the procedure are in particularly strong positions to influence the thinking of age-similar uncircumcised men. Related research points to other categories of persons whose opinions and advice men may hold in especially high regard, such as mothers [24], wives, and other female sexual partners [25–27], and cultural authorities, for example, religious leaders [28]. MMC demand creation messages may be more effective when delivered by important others in men's lives.

Finally, concern over gaps in income associated with recovery from the circumcision procedure highlights the importance of missed work as a barrier to uptake in low resource settings. Elsewhere, unemployed men and informal wage earners have expressed concern about missed work [29]. Our data shows that men with families and agricultural day labourers who rely on their physical strength to generate income are particularly vulnerable to this barrier. The significance of concern over income gaps helps to explain the effectiveness of financial compensation as a demand creation tool [30–32]. Qualitative research investigating how compensation influenced the decision to be circumcised for a Kenyan cohort indicates it (at least partially) offsets lost income as a circumcision barrier, rather than appealing to a motive for financial gain [33]. Our findings corroborate these results.

Qualitative studies have identified barriers and facilitators of MMC uptake across sub-Saharan Africa, including anticipated pain and medical complications [25, 34, 35] cost [36, 37], and perceptions of circumcision's effects on male sexuality [20, 38–42]. Perceived association between circumcision and increased sexual capacity was a concern for some men [40, 41, 43] as was age [34, 44]. These perceptions of MMC also appeared in our data. However, they were overshadowed by predominant themes of expectation of seropositivity and relief at testing, peer support as a mechanism to overcome fear, and concern over income gaps resulting from time away from work.

This study has the following limitations. First, while we believe the insights represented here will be relevant in other settings, as a qualitative study, the results are not formally generalizable. Second, members of support networks were not interviewed as part of the study, precluding an analysis of peer support as a linkage mechanism from the points of view of the peers themselves. Finally, these qualitative data were examined using an inductive, thematic analysis approach. A strength of this approach is that it represents salient aspects of linkage experiences from the points of view of the study participants. A potential disadvantage, in this instance, is that its results largely targeted factors outside the Linkages Study intervention. This limited our ability to evaluate intervention components.

## 5. Conclusions

In addition to the content of MMC demand creation interventions, attention to their circumstances—the “when” and “who” of intervention delivery—may improve effectiveness. The need to sustain income during recovery from circumcision for many men living in settings of economic scarcity must be addressed in order for circumcision targets to be met.

## Figures and Tables

**Table 1:** Sampling of study participants.

Time from enrollment to interview (in months)	
1–3	11 (21.2%)
7–12	15 (28.8%)
>12	26 (50%)
